# Aberrant DNA methylation of *ADAMTS16* in colorectal and other epithelial cancers

**DOI:** 10.1186/s12885-018-4701-2

**Published:** 2018-08-06

**Authors:** Felix Kordowski, Julia Kolarova, Clemens Schafmayer, Stephan Buch, Torsten Goldmann, Sebastian Marwitz, Christian Kugler, Swetlana Scheufele, Volker Gassling, Christopher G. Németh, Mario Brosch, Jochen Hampe, Ralph Lucius, Christian Röder, Holger Kalthoff, Reiner Siebert, Ole Ammerpohl, Karina Reiss

**Affiliations:** 1 Department of Dermatology and Allergology, University Hospital Schleswig-Holstein, University of Kiel, Rosalind-Franklin-Straße 7, 24105 Kiel, Germany; 2 Institute of Human Genetics, University of Kiel, Kiel, Germany; 3 Department of General and Thoracic Surgery, University Hospital Schleswig-Holstein, Kiel, Germany; 4 Medical Department 1, University Hospital Dresden, Technische Universität Dresden, Dresden, Germany; 5 Pathology of the University Medical Center Schleswig-Holstein, Campus Luebeck, Lübeck, Germany; 6 Research Center Borstel, Leibniz Center for Medicine and Biosciences, Borstel, Germany; 7 Thoracic Surgery, LungenClinic Grosshansdorf, Grosshansdorf, Germany; 8 Department of Oral and Maxillofacial Surgery, University of Kiel, Kiel, Germany; 9 Anatomical Institute, University of Kiel, Kiel, Germany; 10 Institute for Experimental Cancer Research, University of Kiel, Kiel, Germany; 11 Institute of Human Genetics, University of Ulm, Ulm, Germany

**Keywords:** Colorectal cancer, ADAMTS16, DNA methylation, Proliferation

## Abstract

**Background:**

ADAMs (a disintegrin and metalloproteinase) have long been associated with tumor progression. Recent findings indicate that members of the closely related ADAMTS (ADAMs with thrombospondin motifs) family are also critically involved in carcinogenesis. Gene silencing through DNA methylation at CpG loci around e.g. transcription start or enhancer sites is a major mechanism in cancer development. Here, we aimed at identifying genes of the ADAM and ADAMTS family showing altered DNA methylation in the development or colorectal cancer (CRC) and other epithelial tumors.

**Methods:**

We investigated potential changes of DNA methylation affecting *ADAM* and *ADAMTS* genes in 117 CRC, 40 lung cancer (LC) and 15 oral squamous-cell carcinoma (SCC) samples. Tumor tissue was analyzed in comparison to adjacent non-malignant tissue of the same patients. The methylation status of 1145 CpGs in 51 *ADAM* and *ADAMTS* genes was measured with the HumanMethylation450 BeadChip Array. ADAMTS16 protein expression was analyzed in CRC samples by immunohistochemistry.

**Results:**

In CRC, we identified 72 CpGs in 18 genes which were significantly affected by hyper- or hypomethylation in the tumor tissue compared to the adjacent non-malignant tissue. While notable/frequent alterations in methylation patterns within *ADAM* genes were not observed, conspicuous changes were found in *ADAMTS16* and *ADAMTS2*. To figure out whether these differences would be CRC specific, additional LC and SCC tissue samples were analyzed. Overall, 78 differentially methylated CpGs were found in LC and 29 in SCC. Strikingly, 8 CpGs located in the *ADAMTS16* gene were commonly differentially methylated in all three cancer entities. Six CpGs in the promoter region were hypermethylated, whereas 2 CpGs in the gene body were hypomethylated indicative of gene silencing. In line with these findings, ADAMTS16 protein was strongly expressed in globlet cells and colonocytes in control tissue but not in CRC samples. Functional in vitro studies using the colorectal carcinoma cell line HT29 revealed that ADAMTS16 expression restrained tumor cell proliferation.

**Conclusions:**

We identified *ADAMTS16* as novel gene with cancer-specific promoter hypermethylation in CRC, LC and SCC patients implicating *ADAMTS16* as potential biomarker for these tumors. Moreover, our results provide evidence that *ADAMTS16* may have tumor suppressor properties.

**Electronic supplementary material:**

The online version of this article (10.1186/s12885-018-4701-2) contains supplementary material, which is available to authorized users.

## Background

Metalloproteinases play important roles in tumor formation and development [1]. Matrix metalloproteases (MMPs) represent the most prominent family associated with tumorigenesis [2]. They are regarded to facilitate tumor progression by degradation of the extracellular matrix (ECM) and by promotion of cancer cell migration. The evolutionarily conserved ADAM (a disintegrin and metalloprotease)-family of cell-bound proteinases mediate the release of cell surface proteins such as growth factors. In particular, ADAM10 and ADAM17 appear to promote cancer progression by releasing HER/EGFR ligands. These proteases are even discussed as potential targets for cancer therapy [3, 4].

Much less is known about the function and relevance of their close relatives, the ADAMTS (ADAMs with thrombospondin motifs) [5]. These secreted proteins share several structural features with MMPs and ADAMs, but are additionally characterized by the presence of thrombospondin motifs which allow them to bind to the ECM. So far, nineteen members of this protease family have been identified in humans [6]. Even though all are presumed to be proteolytically active, many of them are still marked as orphan ADAMTSs without known function or substrate. Some others were found to act as aggrecanases and versicanases and are thus involved in ECM degradation and connective tissue turnover [7, 8].

In recent years, accumulating evidence suggests that ADAM/ADAMTS proteins might play an essentially important role in carcinogenesis [9–12]. This multistep-process involves multiple genetic and epigenetic changes [13], which cause gain of function or activation of oncogenes and loss-of function or inactivation of tumor suppressor genes. Changes in the methylation pattern are a major mechanism controlling the expression and activity of tumor related genes. DNA methylation at promoter and particularly transcription start sites as well as gene body DNA demethylation have been recurrently correlated with inactivation of tumor-suppressor genes [14, 15]. Moreover, such epigenetic changes have been considered promising tools for the early diagnosis of cancer.

While only limited information has been published about potential epigenetic controls of *ADAM* ectodomain sheddases, several *ADAMTS* family members have been described as epigenetic targets and are presumed to act as tumor suppressors. The best described family member *ADAMTS1* was inter alia identified as epigenetically deregulated gene in colorectal and gastric cancer [16–18]. *ADAMTS9* shows high frequency of promoter methylation in esophageal, nasopharyngeal, gastric, colorectal, pancreatic cancer and multiple myeloma [19, 20]. *ADAMTS18* was found to be frequently epigenetically silenced in oesophageal, nasopharyngeal and multiple other carcinomas [21, 22]. ADAMTS16 shows substantial structural similarity to ADAMTS18 [23], however, little is known about its function or regulation [24].

In this study, we report the evaluation of DNA methylation in genes of the *ADAM* and *ADAMTS* families in matched colorectal cancer (CRC), lung cancer (LC) and oral squamous-cell carcinoma (SCC) patient samples. Quite remarkably, *ADAMTS16* promotor hypermethylation was found in all epithelial cancer subtypes analyzed. Moreover, ADAMTS16 protein expression was strikingly decreased in CRC patient samples. Finally, overexpression of ADAMTS16 in HT29 colorectal cancer cells dramatically decreased cell growth. Thus, our data suggest that ADAMTS16 may act as tumor suppressor in certain epithelial cancers.

## Methods

### Patient samples

CRC samples originated from the German National Genome Research Project “Integrated genomic investigation of colorectal carcinoma” were obtained from the Kiel BMB-CCC (biomaterial bank of the Comprehensive Cancer Center, University Hospital of Schleswig-Holstein, Campus Kiel, Germany). The samples were obtained from fresh unfixed surgical resectates, split by pathologists into tumor tissue and adjacent peri-tumoral non-malignant tissue (as controls), and were snap-frozen in liquid N2 and stored in the biobank at − 80 °C until further use. The tissue samples originated from various colon locations. In total, samples from 117 patients were investigated.

Matched LC tissue samples (tumor-free lung and tumor) were obtained from patients undergoing pneumectomy or lobectomy at the LungenClinic Grosshansdorf, Germany (*n* = 40) in the course of surgical treatment of previously diagnosed lung cancer.

Native tissue samples from patients suffering from oral lichen planus and/or oral squamous-cell carcinoma (*n* = 15) were collected from consultation hours for oral mucosa at the Department of Cranio-Maxillofacial Surgery, University Hospital of Schleswig-Holstein, Kiel Campus, Kiel, Germany. As control samples, non-inflamed tissue from the same patient was collected.

### DNA methylation analysis

Genomic DNA extraction was done using DNeasy kit (Qiagen, Germany). DNA samples were bisulfite converted with the EZ DNA Methylation™ Kit (Zymo Research Corporation, USA) and afterwards measured for DNA methylation with the Infinium Human Methylation 450 k BeadChip (Illumina Inc., USA) according to the manufacturer’s protocol. The generated IDAT files were further processed with the Genome Studio Software (version 2011.1; Methylation Analysis Module version 1.9.0, Illumina) to derive the β-values. Thereby internal array controls and the default settings were used for data normalization. Methylation levels in Illumina Methylation assays are quantified using the ratio of intensities between methylated and unmethylated alleles. The β-values are continuous and range from 0 (unmethylated) to 1 (completely methylated) [25].

### Cell culture and transfection

Mycoplasma free HT29 cells were purchased from the American Type Culture Collection (ATCC), and grown in high glucose DMEM (Thermo Fisher Scientific) supplemented with 10% fetal calf serum (FCS) and 1% penicillin/streptomycin (Pen/Strep). Cells were transfected using Turbofect Transfection Reagent (Thermo Fisher Scientific) according to the manufacturer’s instructions. 24 h after transfection, cells were transferred to the X-Celligence device and in parallel approaches harvested for immunoblot analysis.

### Impedance based xCELLigence proliferation assay

The xCELLigence invasion assay (ACEA Biosciences, USA) is based on changes in electrical impedance at the interphase between cell and electrode as migrating cells move through a barrier. These changes can be directly correlated with the proliferative capacity of seeded cells. The technique provides an advantage over existing standard proliferation assays, since the data is obtained continuously in real-time, when compared to end-point analysis in other methods. To analyze cell proliferation, HT29 cells were seeded at a density of 20,000 cells/well on E16 plates. The impedance value of each well was automatically monitored by the xCELLigence system for duration of 24 h and expressed as a CI (cell index) value. Averages of duplicates are shown derived from three independent experiments. The rate of cell growth was determined by calculating the slope of the line between the starting point and 24 h.

### Western blot analysis

Cells were washed once with PBS and lysed in lysis buffer (5 mM Tris-HCl (pH 7.5), 1 mM EGTA, 250 mM saccharose, 1% Triton X-100) supplemented with cOmplete inhibitor cocktail (Roche Applied Science) and 10 mM 1,10-phenantroline monohydrate. Equal amounts of protein were loaded on 10% SDS-PAGE gels. The samples were electrotransferred onto polyvinylidene difluoride membranes (Hybond-P; Amersham) and blocked overnight with 5% skim milk in Tris-buffered saline (TBS). After incubation with anti-ADAMTS16 antibody (Santa Cruz, sc-50,490) in blocking buffer, the membranes were washed three times in TBST (TBS containing 0.1% Tween-20). Primary antibody was detected using affinity-purified peroxidase (POD)-conjugated secondary antibody (1:10,000) for 1 h at room temperature. Detection was carried out using the ECL detection system (Amersham). Signals were recorded by a luminescent image analyzer (Fusion FX7 imaging system; PEQLAB Biotechnologie). Equal loading as well as efficiency of transfer were routinely verified by reprobing the membrane for tubulin (DSHB clone E7).

### Immunohistochemistry

Cryosections (7 μm) of the CRC samples were fixed with acetone. Slides were incubated in 3% H_2_O_2_ in PBS for 30 min. After blocking of the nonspecific binding (0.75% BSA in PBS), the sections were incubated with anti-ADAMTS16 antibody (Origene, dilution 1:100) over night. The staining was visualized by peroxidase-conjugated secondary antibody and diaminobenzidine (Vector labs). Finally, sections were counterstained by hemalum and embedded in Kaiser’s glycerol gelatine and photographed with an Axioplan microscope (Zeiss, Germany). The corresponding negative controls were stained omitting the anti-ADAMTS16 antibody.

### Statistical analysis

Comparison of the DNA methylation status of patient matched tumor and peritumoral non-malignant DNA samples was performed using the script language R 3.2.2 (R foundation), Graphpad Prism 5.04 (GraphPad Software Inc., USA) and Excel 2010 (Microsoft, USA). CpGs were defined as differentially methylated if the difference of the mean β-values (∆βmean) was larger than 0.2 (|∆βmean| ≥ 0.2) compared to the control and significant after Wilcoxon signed-rank testing with Benjamini-Hochberg multiple testing correction for the 1145 tests performed (*P* < 0.05). CpGs which did not meet these criteria, but showed a methylation difference of 0.1 ≤ |∆βmean| < 0.2 (*P* < 0.05) were defined as intermediate methylated.

## Results

### Major DNA methylation changes in the *ADAMTS16* gene in CRC

The methylation status of 1145 CpGs in 51 ADAM and ADAMTS genes was analyzed with the HumanMethylation450 BeadChip Array. With this BeadChip Array the methylation in 485,577 positions can be analyzed (CpG, non-CpG and SNP positions). Of these, we analyzed all CpGs with annotation to ADAM and ADAMTS genes (annotation by Illumina). CpGs were defined as differentially methylated if the difference of the mean β-values (∆βmean) was larger than 0.2 (|∆βmean| ≥ 0.2) compared to the control and significant after Wilcoxon signed-rank testing with multiple testing correction (*P* < 0.05). In first analyses, tissues from 117 colorectal cancer (CRC) patients were studied. Resected samples of the tumor tissue and, as control, peri-tumoral non-malignant tissue of the same patient were analyzed for methylation differences.

**Fig. 1:**
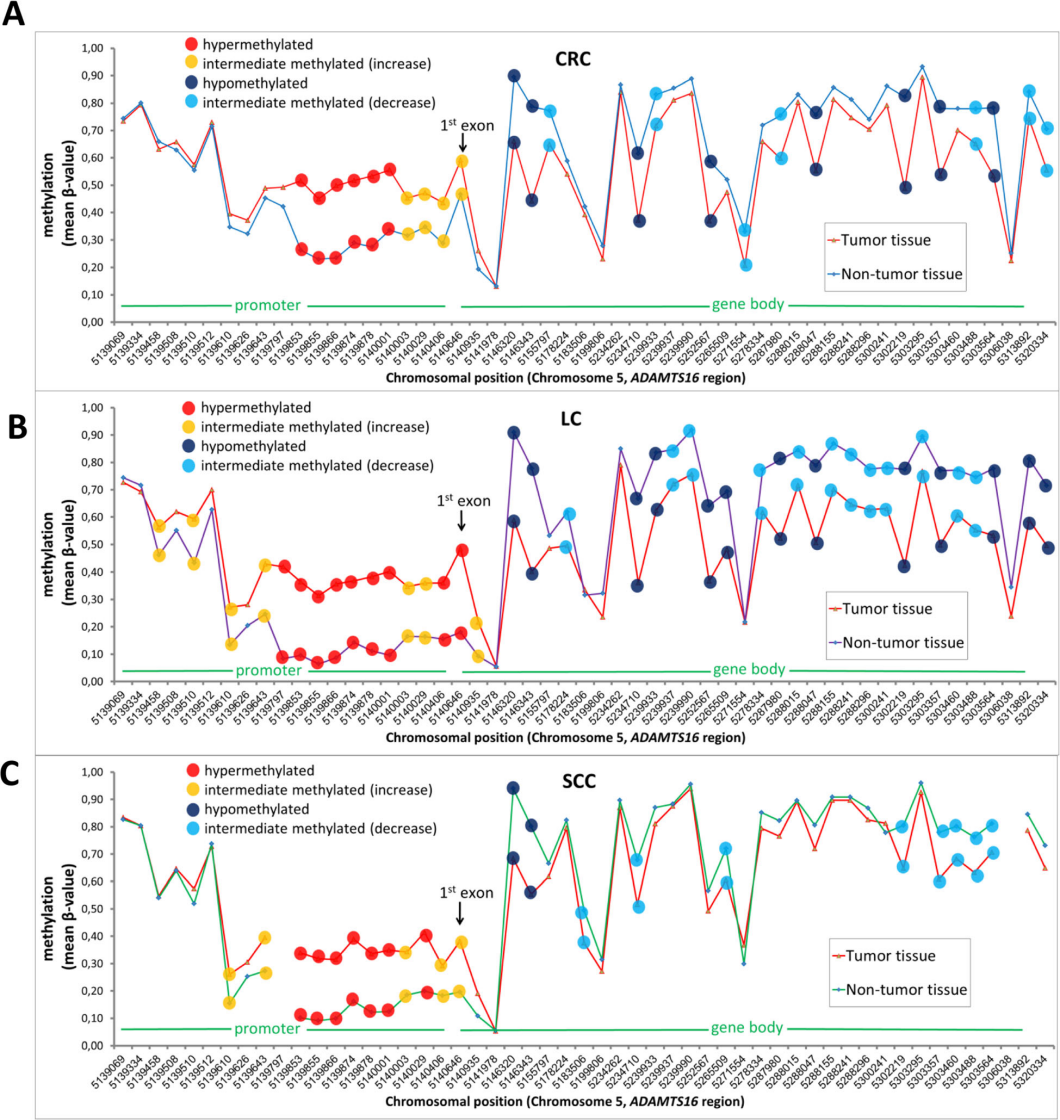
Methylation profile of the *ADAMTS16* gene in **a** colorectal cancer (CRC), **b** lung cancer (LC) and **c** oral squamous-cell carcinoma (SCC) patients. Shown is the average methylation (mean β-value) of 53 different CpG sites in *ADAMTS16***.** All three cancer entities show very similar methylations profiles. Hypermethylation was defined as ∆β_mean_ ≥ 0.2 (*P* < 0.05), hypomethylation as ∆β_mean_ ≤ − 0.2 (*P* < 0.05) and intermediate methylation as 0.1 ≤ |∆β_mean_| < 0.2 compared to the control (*n* = 117 (CRC), *n* = 40 (LC), *n* = 15 (SCC))

A total of 72 CpGs in 18 genes were significantly affected by hyper- or hypomethylation (more than 20% difference) (Additional file 1: Figure S1). *ADAM12* was the only member of the *ADAM* family showing noteworthy methylation changes. In contrast, several *ADAMTS* family member were affected. Here, the most striking methylation changes were located in the *ADAMTS16* and *ADAMTS2* gene. In both genes, 14 CpGs were found to be differentially methylated (|∆βmean| ≥ 0.2, P < 0.05). The methylation status of all *ADAMTS16* CpGs in CRC patients is shown as (Additional file 1: Figure S2). Six CpGs in the promoter region were found to be hypermethylated, while eight CpGs were hypomethylated in the gene body. Additionally, 11 CpGs (together 47.2% of all CpGs) showed an intermediate methylation difference of more than 0.1 (0.1 ≤ |∆βmean| < 0.2, P < 0.05). The methylation profile of *ADAMTS16* in CRC is depicted in Fig. 1a.

### The changes in *ADAMTS16* DNA methylation show a common pattern in three different epithelial cancers

**Fig. 2:**
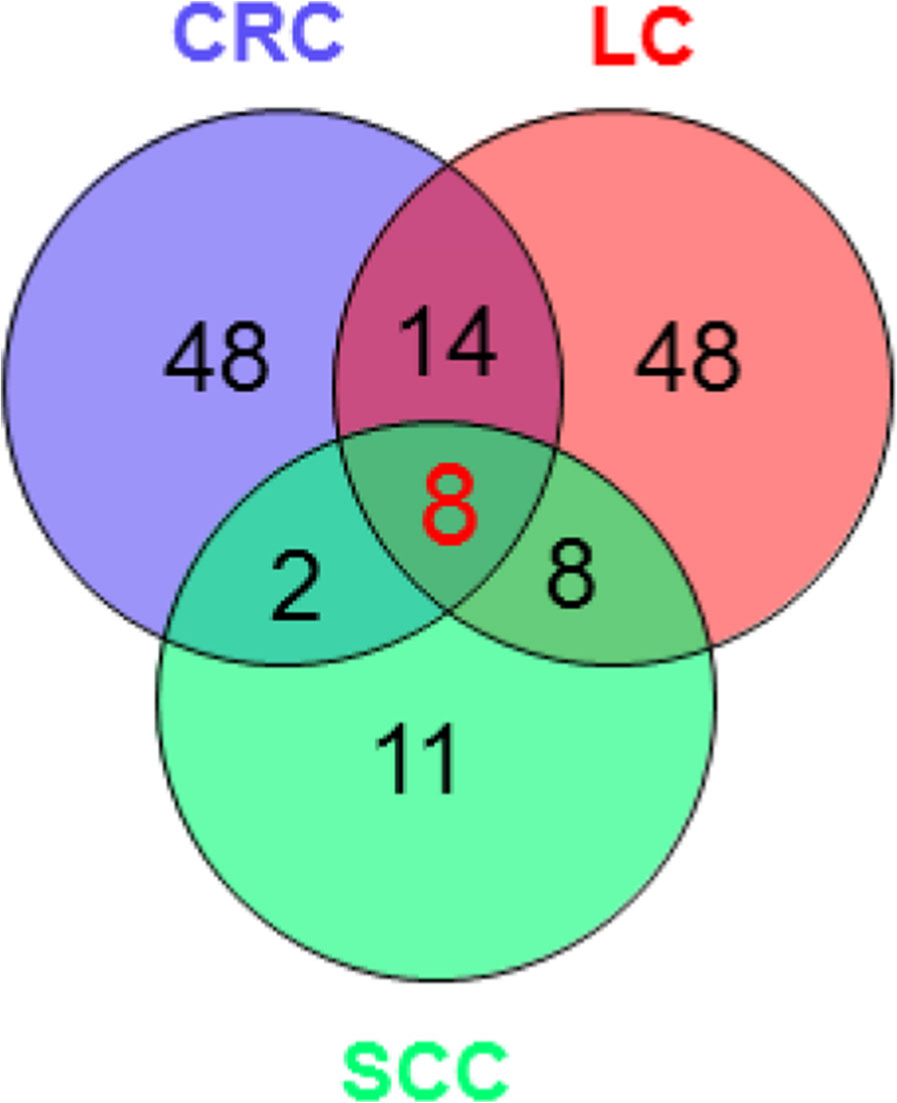
Overlap of differentially methylated CpGs in lung cancer (LC), colorectal cancer (CRC) and oral squamous-cell carcinoma (SCC). 8 CpGs are commonly differentially methylated in the three cancer entities. All are located in the *ADAMTS16* gene. The venn diagram was generated with VENNY 2.0 (Oliveros, 2007)

To delineate whether the observed epigenetic alterations in the *ADAMTS16* gene in colorectal cancer were CRC specific, samples of two other epithelial cancers were investigated of all ADAMs and ADAMTS genes. Resectates from 40 lung cancer (LC) and 15 oral squamous-cell carcinoma (SCC) patients were analyzed for methylation changes. A total of 78 differentially methylated CpGs were found in LC and 29 in SCC. Again, only few members of the *ADAM* family showed methylation changes and these were rather inconspicuous. No differential methylation was found for *ADAM12* and only one single change was detected for *ADAMTS2*. Strikingly, 8 CpGs in all three cancer entities showed a similar methylation pattern. All of them were located in the *ADAMTS16* gene (Table 1). In Fig. 2, the Venn diagram depicts the overlap of the differentially methylated CpGs between LC, CRC and SCC.

**Table 1:**
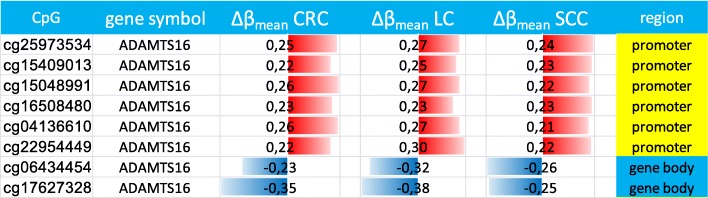
Common differentially methylated CpGs in CRC, LC and SCC

The difference between the average DNA methylation of the control and the cancer tissues (∆β_mean_) of the 8 commonly differentially methylated CpGs. All are located in the *ADAMTS16* gene**.** CpGs were defined as differentially methylated if the |∆β_mean_| in the cancer samples (canc) was > 0.2 compared to the control (ctrl); (n = 117 (CRC), n = 40 (LC), n = 15 (SCC)). The colored bars represent the magnitude of hypermethylation (red) or hypomethylation (blue)

**Fig. 3:**
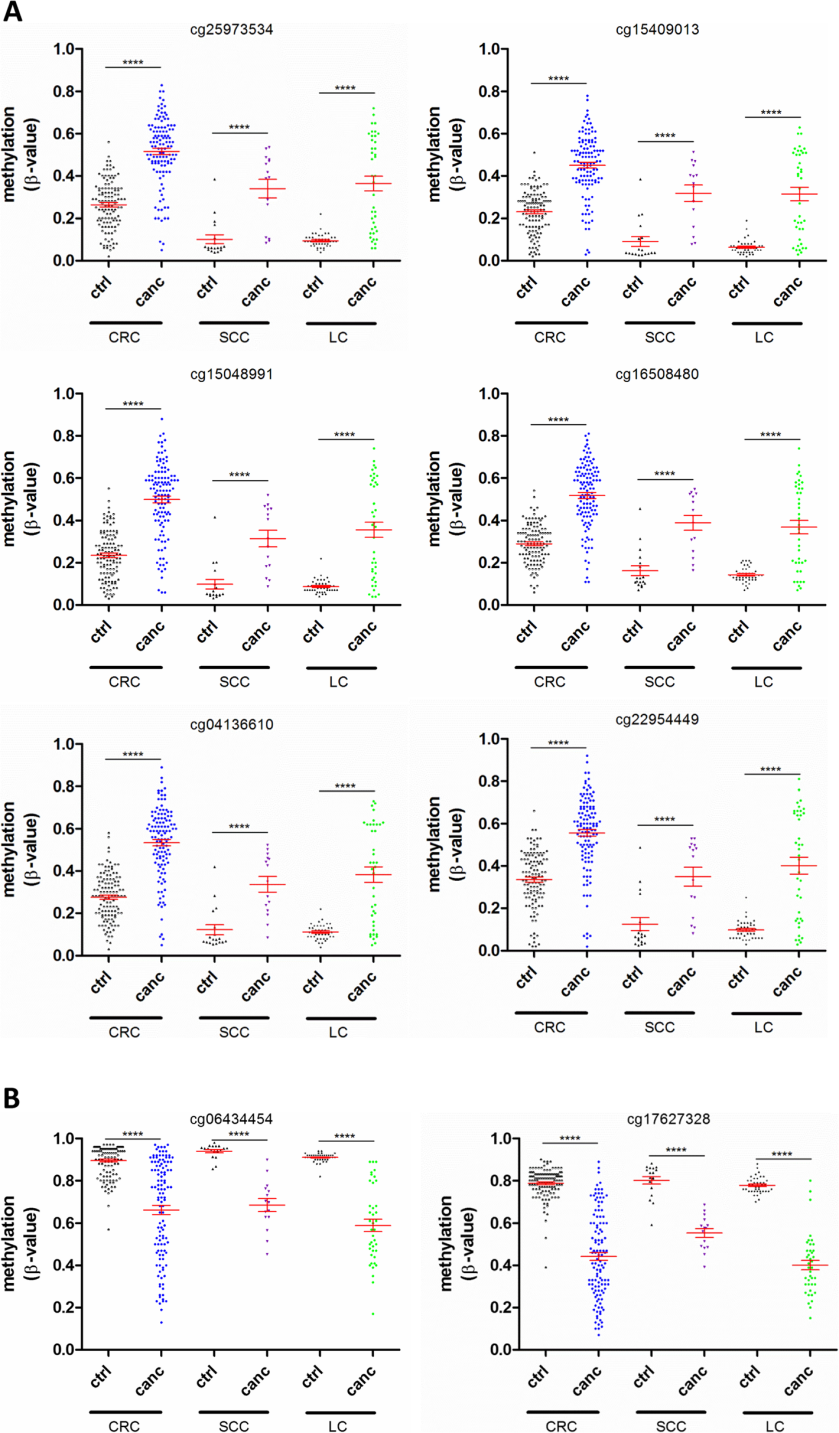
Comparision of hyper/hypomethylated *ADAMTS16* CpGs in colorectal cancer (CRC), lung cancer (LC) and oral squamous-cell carcinoma (SCC) patients. **a** Six hypermethylated *ADAMTS16* CpGs in CRC patients were also hypermethylated in LC and SCC patients. Data represent the methylation (β-value) for individual patients (spots) with the mean ± SEM (red lines). Data were statistically analyzed with Wilcoxon signed-rank test and corrected for multiple testing with Benjamini-Hochberg method (**** *P* < 0.0001, (*n* = 117 (CRC), *n* = 40 (LC), *n* = 15 (SCC)). ctrl = peri-tumoral non-malignant tissue; canc = cancer tissue; SEM = standard error of mean. **b** Two hypomethylated *ADAMTS16 CpGs* in CRC patients are also hypomethylated in LC and SCC patients. Data represent the methylation (β-value) for individual patients (spots) with the mean ± SEM (red lines). Data were statistically analyzed with Wilcoxon signed-rank test and corrected for multiple testing with Benjamini-Hochberg method (**** *P* < 0.0001, (*n* = 117 (CRC), *n* = 40 (LC), *n* = 15 (SCC)). ctrl = peri-tumoral non-malignant tissue; canc = cancer tissue; SEM = standard error of mean

The methylation profiles of the LC and SCC cancer entities for *ADAMTS16* are depicted in Fig. 1b and c. The overall methylation profiles and methylation changes were extremely similar in all three cancer entities. Six CpGs in the promoter region immediately 5′ of the transcription start site were commonly hypermethylated, whereas two CpGs in the gene body of *ADAMTS16* were hypomethylated compared to the control. Furthermore, the overall pattern of the graphs was very similar reflecting a similar *ADAMTS16* methylation profile in these three cancer entities. A direct comparison of these 8 CpGs is shown in Fig. 3. It revealed that the direction change was the same in all three cancer entities. The 6 CpGs in the promoter region were all hypermethylated, whereas the 2 CpGs in the gene body of *ADAMTS16* were hypomethylated as compared to the control. The mean methylation of these CpGs in lung cancer and oral squamous-cell carcinoma patients was comparable. In contrast, CRC tissues tended to a higher mean methylation than LC and SCC.

### ADAMTS16 protein expression is decreased in colorectal cancer tissue

**Fig. 4:**
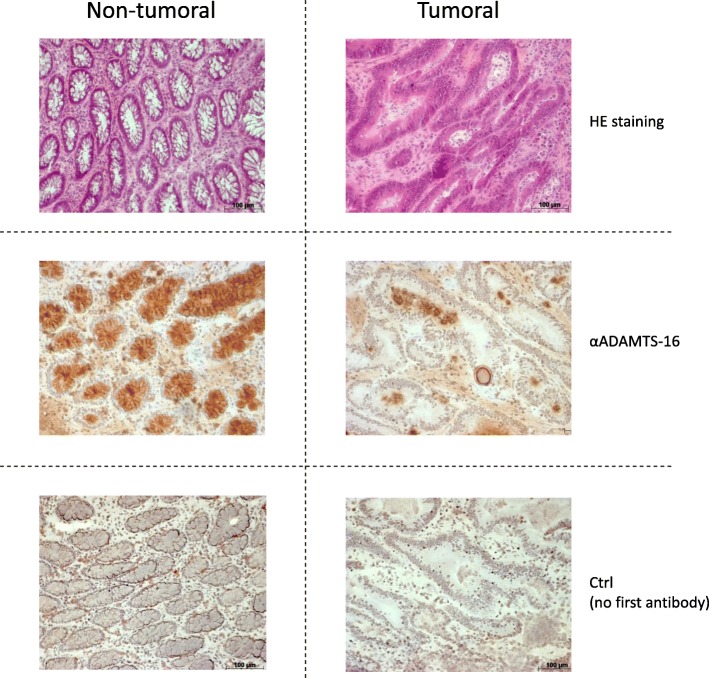
ADAMTS16 expression in normal and colorectal tissue. ADAMTS16 protein expression was analyzed in CRC and control samples of the same patients by immunohistochemistry. In normal tissue (NT) ADAMTS16 showed a strong expression in the epithelial cells of the crypts. This staining was severely reduced in tumorous tissue. Representative images of one out of 10 patients of the study population. Scale Bar = 100 μm

Next, we examined ADAMTS16 protein expression by immunohistochemical staining. Corresponding non-tumor and tumor tissue samples of ten patients of the CRC study population were analyzed. In all control tissues, a strong ADAMTS16 staining was found in the colorectal epithelium (Fig. 4). In particular, the goblet cells and colonocytes lining the crypts showed a strong protein expression. In contrast, in all tumor tissues no or only very weak immunoreactivity was observed.

### Overexpression of ADAMTS16 impairs tumor cell proliferation

**Fig. 5:**
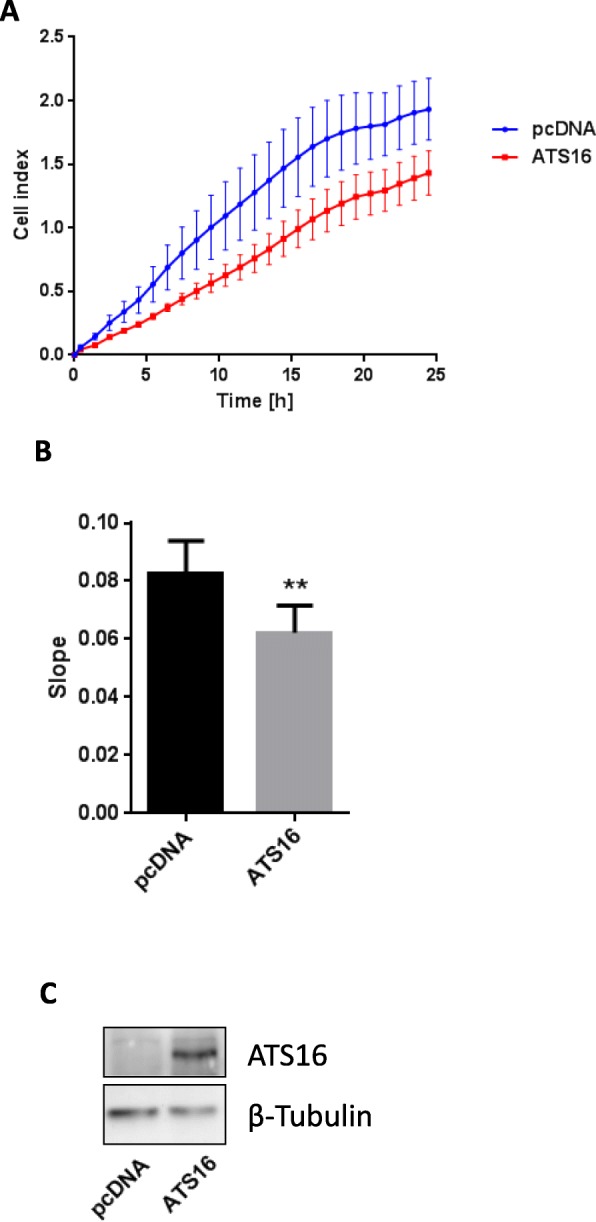
ADAMTS16 overexpression reduces cell proliferation of HT29 cells. **a** Proliferation of human colorectal adenocarcinoma HT29 cells was measured continuously as cell index using the xCELLigence system. **b** The slope of the growth curve was calculated and found to be significant diminished upon ADAMTS16 (ATS16) overexpression compared to mock (pcDNA) transfected cells. Experiments were performed in duplicates. Mean ± SEM, (*n* = 3). *indicates significant difference (*p* < 0.05, ANOVA). **c** Anti-ADAMTS16 Western blot of mock-transfected HT29 cells, and cells transfected with ADAMTS16, indicating successful transfection. β-tubulin was used as loading control

The human colorectal adenocarcinoma cell line HT29 was used for the analysis of ADAMTS16 function. Proliferation of HT29 cells was measured continuously in real time using the xCELLigence system (Fig. 5a). Overexpression of ADAMTS16 resulted in impaired cell proliferation. To further emphasize this, we calculated the slope of the growth curve, which was significantly decreased upon ADAMTS16 transfection (Fig. 5b). ADAMTS16 transfection efficiency was controlled by immunoblotting (Fig. 5c). These results support the assumption that ADAMTS16 could act as a tumor suppressor.

## Discussion

CpG promoter hypermethylation has been demonstrated to be a frequent event during carcinogenesis. In this study, we aimed to find out whether members of the *ADAM* and *ADAMTS* family might represent novel gene targets epigenetically inactivated in epithelial tumorigenesis. Comparing malignant and non-malignant tissues of the same patients, we identified *ADAMTS16* as a gene with cancer-specific promoter hypermethylation in CRC, LC and SCC patients.

Several ADAM family members, particularly ADAM9, ADAM10, ADAM12, ADAM15 and ADAM17, have been implicated in cancer formation and progression. ADAM10 and ADAM17 are even discussed as potential targets for cancer therapy [3]. However, except for *ADAM12*, we did not find relevant changes in the DNA methylation pattern in any of these tumor-associated proteases. The changes observed for *ADAM12* were located in the gene body and only found in CRC but not in SCC or LC patients. Overall, our findings indicate that differences in gene DNA methylation are unlikely to be responsible for the control of ADAM function in tumors. Instead, these enzymes seem to be rather controlled by posttranslational mechanisms. This assumption is in accordance with recent data stressing the relevance of protein maturation, localization and cell membrane changes for protease activation [26, 27].

In contrast to the *ADAM* family, epigenetic silencing and genetic inactivation in *ADAMTS* family members has been frequently reported. This observation led to the concept that these protease family members could be important tumor suppressors. *ADAMTS15* is genetically silenced in human colorectal cancer [28]. *ADAMTS1* and *ADAMTS9* have been found to be epigenetically silenced in diverse malignant tumors [16, 19]. *ADAMTS12* has been identified as potential tumor suppressor in colorectal cancer [29]. *ADAMTS8* was shown to be differentially methylated in brain, thyroid, lung, nasopharyngeal, esophageal, gastric and colorectal cancers [30]. Also *ADAMTS18* has recently been identified as tumor suppressor gene. Differential methylation has been reported in renal, gastric, colorectal, pancreatic, esophageal, and nasopharyngeal carcinomas [21, 22].

ADAMTS16 shares conspicuous structural similarity with ADAMTS18 [23]. However, ADAMTS16 is one of the least examined proteins from the whole ADAMTS family and little is known about its function. Today, the only known substrate of ADAMTS16 is α2-macroglobulin [31], a general inhibitor of proteases. In this context, an involvement in the human ovarian follicle maturation has been proposed [32]. The role of ADAMTS16 in tumorigenesis is not clear. So far, no epigenetic modifications have ever been reported for this protease.

Here, we identified *ADAMTS16* as commonly differentially methylated gene in three different types of epithelial cancers. *ADAMTS16* promoter hypermethylation at six CpGs immediately upstream of the transcription start site and hypomethylation in two CpGs in the gene body is very suggestive of decreased protein expression. To establish whether this would be the case, we analyzed CRC tumors and non-tumorous patient samples via immunohistochemistry. These analyses revealed that expression of ADAMTS16 is markedly decreased in CRC. The possibility that this might be causally linked to CpG-hypermethylation within the promoter region was supported through analysis of data provided by The Cancer Genome Atlas (TCGA, http://cancergenome.nih.gov/, accessed on 05.02.2015) for a colon adenocarcinoma and rectum adenocarcinoma cohort (COADREAD, *n* = 44 (ctrl), *n* = 384 (canc)). These data are based on non-matched control and cancer samples. Gratifyingly, the same methylation changes in the 8 commonly differentially methylated CpGs that we described for CRC, LC and SCC patients were found. Gene expression analysis for the same TCGA COADREAD cohort (*n* = 22 (ctrl), *n* = 224 (canc)) revealed that *ADAMTS16* mRNA expression was significantly decreased from 0.29 in the control (ctrl) to 0.04 in the cancer tissue (canc) (*P* < 0.0001). This decrease reflects a reduction of the *ADAMTS16* mRNA expression of 86.3%.

It became of immediate interest to investigate whether expression of ADAMTS16 might impact on a cellular function linked to carcinogenesis. Assessment of cell proliferation was chosen as a first approach in this direction. Overexpression of ADAMTS16 in HT29 colorectal cancer cells significantly reduced cell proliferation. These data are in accordance with data by Surridge et al., who showed that overexpression of ADAMTS16 in chondrosarcoma cells led to a decrease in cell proliferation and migration [24]. However, further analyses of the ADAMTS16 effects on tumor cell migration and invasion are warranted in order to find out whether *ADAMTS16* might represent a novel tumor suppressor gene for CRC, LC and SCC.

## Conclusions

In summary, our data identify *ADAMTS16* as common differentially methylated gene in CRC, LC and SCC patients. Epigenetic changes in DNA methylation possibly lead to down-regulation of ADAMTS16-expression that may be causally linked to development of CRC. Our investigation leads to the tentative conclusion that ADAMTS16 may exert an anti-proliferative function through mechanisms that require future resolution. Further epigenetic analyses of epithelial tumors and functional studies characterising ADAMTS16 are warranted.

## Additional file

Additional file 1: **Figure S1.** Differentially methylated CpGs in tumor tissue compared to non-tumor tissue in CRC patients. Tumor resectats (canc) and peri-tumoral non-malignant resectats (ctrl) from the same patient were analyzed with the HumanMethylation450 BeadChip Array for the methylation of 450 k CpG sites. 72 of 1145 CpGs located in ADAM/TS genes were differentially methylated. The depicted β-value represents a quantitation of the methylation level of the respective CpG-locus. Data were statistically analyzed with Wilcoxon signed-rank and corrected for multiple testing with Benjamini-Hochberg method (**** *P* < 0.0001). Hypermethylation was defined as ∆βmean ≥0.2 (*P* < 0.05) and hypomethylation as ∆βmean ≤ − 0.2 (*P* < 0.05) compared to the control. Only hyper- or hypomethylated CpGs are presented. *p*-values were rounded to the 5th decimal place where applicable. The colored bars represent the magnitude of hypermethylation (red), hypomethylation (blue) or the absolute value of the methylation change (green). **Figure S2.** Methylation status of all ADAMTS16 CpGs in CRC patients. Tumor resectats (*n* = 117, canc) and peri-tumoral non-malignant tissue (n = 117, ctrl) from the same patient were analyzed with the HumanMethylation450 BeadChip Array for the methylation of 450 k CpG sites. In ADAMTS16, 14 out of 53 CpGs were differentially methylated and 11 CpGs showed intermediate methylation alterations (0.1 ≤ |∆βmean| < 0.2). The depicted β-value represents a quantitation of the methylation level of the respective CpG-locus. Data were statistically analyzed with Wilcoxon signed-rank test and corrected for multiple testing with Benjamini-Hochberg method (* *P* < 0.05, ** *P* < 0.01, *** *P* < 0.001, **** P < 0.0001). Hypermethylation was defined as ∆βmean ≥0.2 (P < 0.05) and hypomethylation as ∆βmean ≤ − 0.2 (*P* < 0.05) compared to the control. Ctrl = control, peri-tumoral non-malignant tissue; canc = cancerous tissue. *p*-values were rounded to the 6th decimal place where applicable. The colored bars represent the magnitude of hypermethylation (red), hypomethylation (blue) or the absolute value of the methylation change (green) (DOCX 491 kb)

## Abbreviations

ADAM: a disintegrin and metalloproteinase
ADAMTS: a disintegrin and metalloproteinase domain with thrombospondin motifs
canc: cancer
CRC: colorectal cancer
Ctrl: control
ECM: extracellular matrix
LC: lung cancer
MMPs: matrix metalloproteases
SCC: oral squamous-cell carcinoma
