# Supplementary figures and images for: Aberrant DNA methylation of ADAMTS16 in colorectal and other epithelial cancers
Source: BMC Cancer. 2018 Aug 6;18:796. doi: 10.1186/s12885-018-4701-2 (PMC6080380; doi:10.1186/s12885-018-4701-2)

Supplementary Figure 1
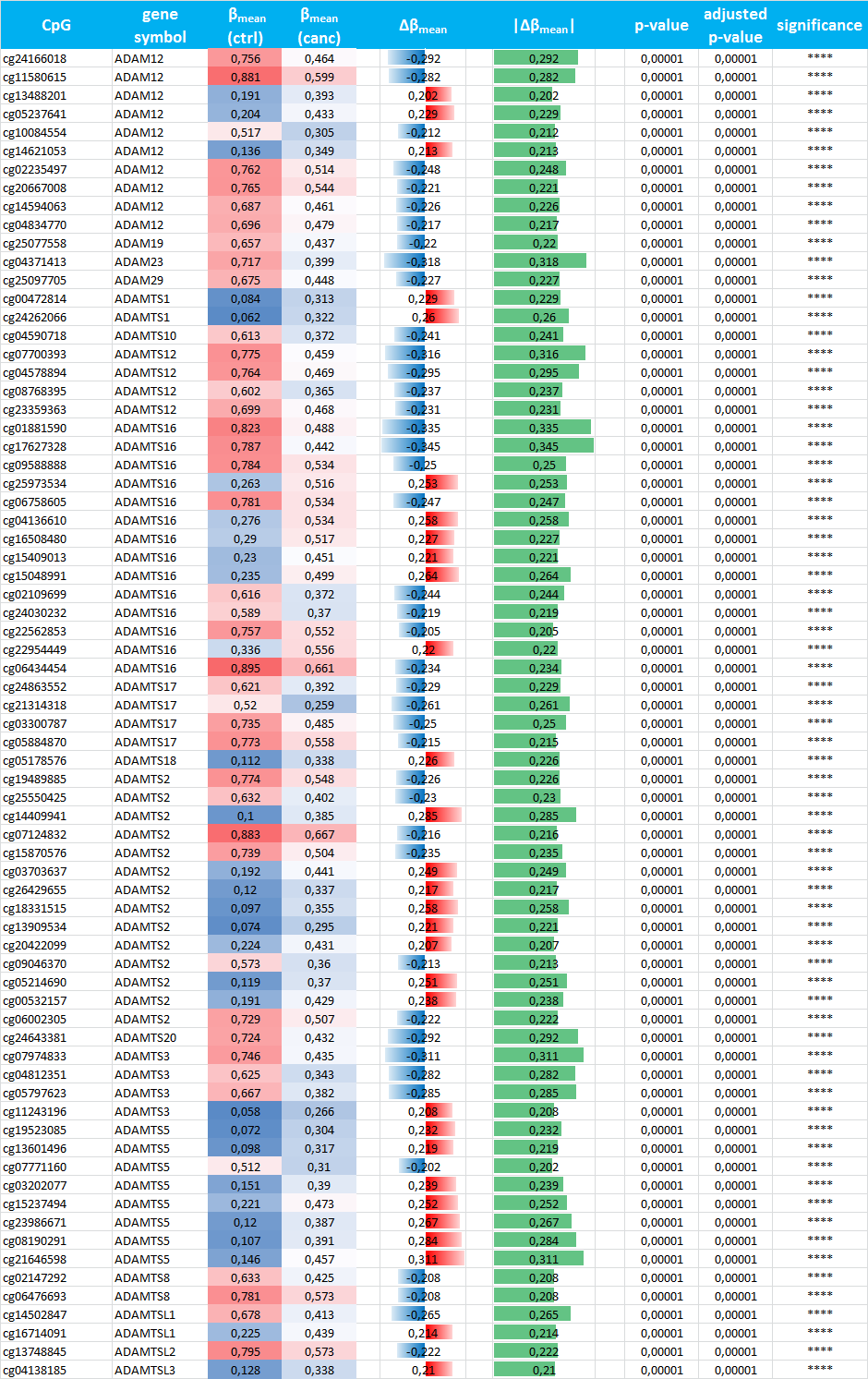


Supplementary Figure 2
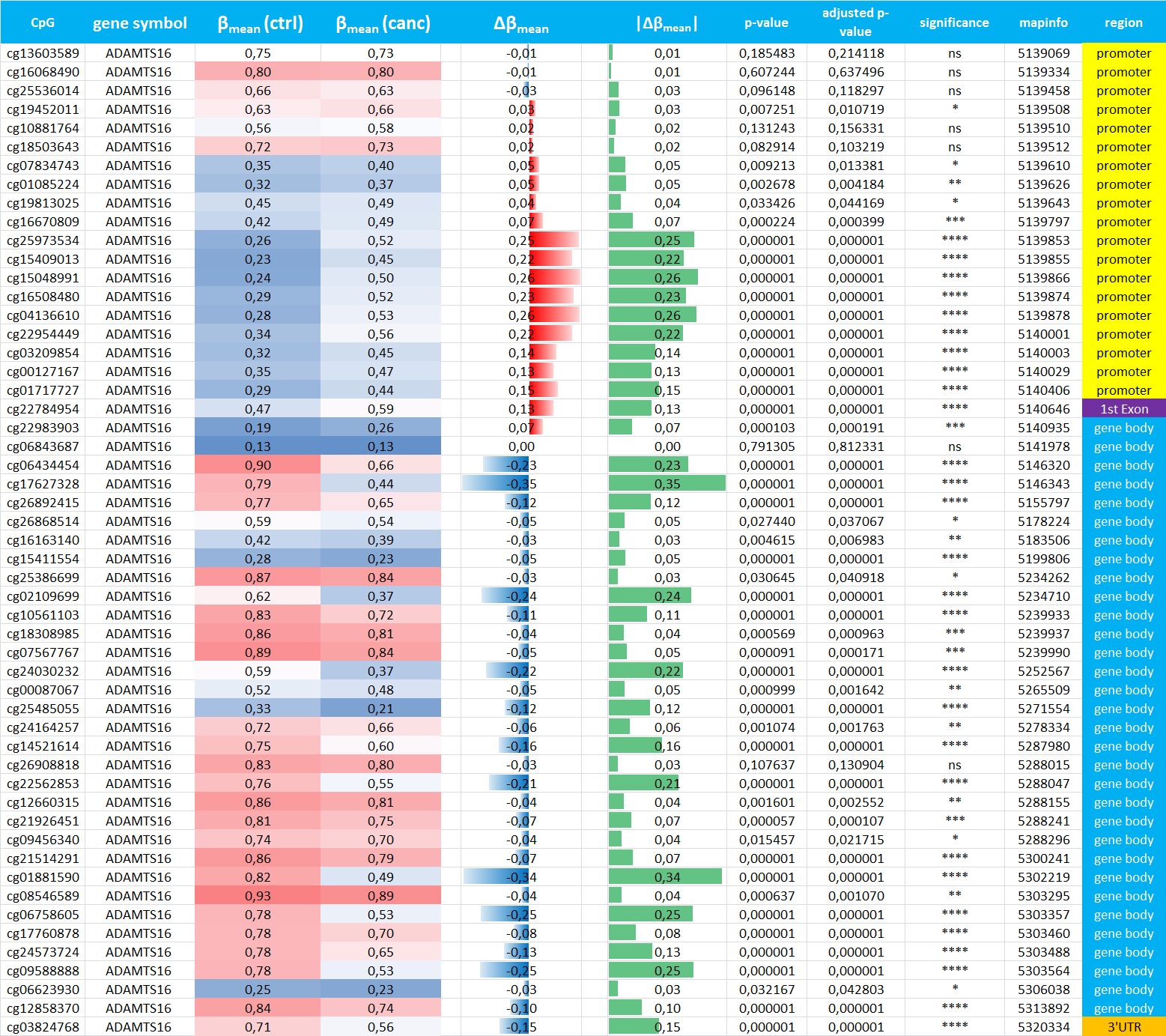


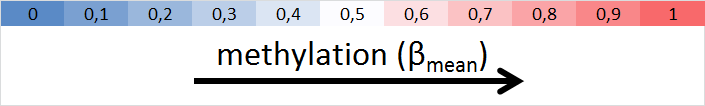

Supplement: Supplementary file 1 — Figure S1. Differentially methylated CpGs in tumor tissue compared to non-tumor tissue in CRC patients. Tumor resectats (canc) and peri-tumoral non-malignant resectats (ctrl) from the same patient were analyzed with the HumanMethylation450 BeadChip Array for the methylation of 450 k CpG sites. 72 of 1145 CpGs located in ADAM/TS genes were differentially methylated. The depicted β-value represents a quantitation of the methylation level of the respective CpG-locus. Data were statistically analyzed with Wilcoxon signed-rank and corrected for multiple testing with Benjamini-Hochberg method (**** P < 0.0001). Hypermethylation was defined as ∆βmean ≥0.2 (P < 0.05) and hypomethylation as ∆βmean ≤ − 0.2 (P < 0.05) compared to the control. Only hyper- or hypomethylated CpGs are presented. p-values were rounded to the 5th decimal place where applicable. The colored bars represent the magnitude of hypermethylation (red), hypomethylation (blue) or the absolute value of the methylation change (green). Figure S2. Methylation status of all ADAMTS16 CpGs in CRC patients. Tumor resectats (n = 117, canc) and peri-tumoral non-malignant tissue (n = 117, ctrl) from the same patient were analyzed with the HumanMethylation450 BeadChip Array for the methylation of 450 k CpG sites. In ADAMTS16, 14 out of 53 CpGs were differentially methylated and 11 CpGs showed intermediate methylation alterations (0.1 ≤ |∆βmean| < 0.2). The depicted β-value represents a quantitation of the methylation level of the respective CpG-locus. Data were statistically analyzed with Wilcoxon signed-rank test and corrected for multiple testing with Benjamini-Hochberg method (* P < 0.05, ** P < 0.01, *** P < 0.001, **** P < 0.0001). Hypermethylation was defined as ∆βmean ≥0.2 (P < 0.05) and hypomethylation as ∆βmean ≤ − 0.2 (P < 0.05) compared to the control. Ctrl = control, peri-tumoral non-malignant tissue; canc = cancerous tissue. p-values were rounded to the 6th decimal place where applicable. The colored bars re [file 12885_2018_4701_MOESM1_ESM.docx]
